# Packaging-based visual-storytelling nudge to improve antibiotic adherence in uncomplicated urinary tract infection: a pilot randomized trial in Pakistan

**DOI:** 10.1186/s12879-025-12246-x

**Published:** 2025-12-01

**Authors:** Iltaf Hussain, Sen Xu, Yi Dong, Muhammad Fawad Rasool, Jamshid Ullah, Amjad Khan, Muhtar Kadirhaz, Miaomiao Xu, Chengzhou Tang, Wei Zhao, Faiz Ullah Khan, Jie Chang, Yu Fang

**Affiliations:** 1 Department of Pharmacy Administration, School of Pharmacy, Xi’an Jiaotong University, Xi’an, China; 2 Center for Drug Safety and Policy Research, Xi’an Jiaotong University, Xi’an, China; 3 Shaanxi Center for Health Reform and Development Research, Xi’an, China; 4 Research Institute for Drug Safety and Monitoring, Institute of Pharmaceutical Science and Technology, Western China Science and Technology Innovation Harbor, Xi’an, China; 5 Department of Pharmacy Practice, Faculty of Pharmacy, Bahauddin Zakeriya University, Multan, Pakistan; 6 Department of Medical Laboratory Technology, Institute of Paramedical Sciences, Khyber Medical University, Peshawar, Khyber Pakhtunkhwa Pakistan; 7 Department of Pharmacy, The First Affiliated Hospital, Xi’an Jiaotong University, Xi’an, China; 8 Department of Pharmacy, Quaid-i-Azam University, Islamabad, Pakistan; 9 Department of Pharmacy Administration and Clinical Pharmacy, School of Pharmaceutical Sciences, Peking University Health Science Center, Peking University, Beijing, China; 10 International Research Center for Medicinal Administration (IRCMA), Peking University, Beijing, China

**Keywords:** Nudge intervention, Antibiotic adherence, Pilot study

## Abstract

**Background:**

Antibiotic resistance (ABR) is a growing public health issue, where non-adherence to antibiotics accelerates the development of resistance. Packaging-based nudge interventions may offer a low-cost and scalable solution. This pilot randomized controlled trial (RCT) assessed the feasibility of a visual storytelling sticker affixed to antibiotic packaging to improve antibiotic adherence in patients with uncomplicated urinary tract infections (UTIs).

**Methods:**

A parallel two-arm pilot RCT was conducted across six tertiary-care hospitals between May and July 2025. Physician-confirmed uncomplicated UTI patients, prescribed with oral antibiotics, were randomized in a 1:1 manner to receive either antibiotics with a visual storytelling sticker (intervention) or standard care (control). Primary outcomes were feasibility metrics, including recruitment, retention, and intervention delivery fidelity. Antibiotic adherence, measured by pill count at the completion of treatment, was assessed as an exploratory outcome.

**Results:**

Of the 120 patients approached, 114 were screened, and 84 were deemed eligible. Seventy patients consented, of whom 66 were randomized (33 per arm). The recruitment and retention rates were 61.4% and 84.8%, respectively, whereas the intervention delivery fidelity was 87.5%. Exploratory analysis showed a 14.3% improvement in adherence in the intervention group (63.3%, 95%CI 61.1–65.5) compared with the control group (49%, 95%CI 46.5–51.5), though the study was not powered to test effectiveness.

**Conclusions:**

This pilot trial showed that a packaging-based visual storytelling sticker can be successfully integrated into antibiotic dispensing workflows. Preliminary adherence differences offer estimates to inform sample size and design of a larger, definitive RCT.

**Trial registration:**

Chinese Clinical Trial Registry (ChiCTR), ChiCTR2500111346. Registered on 30 October 2025. Retrospectively registered.

**Supplementary Information:**

The online version contains supplementary material available at 10.1186/s12879-025-12246-x.

## Strengths and limitations

A pilot randomized trial was conducted to evaluate the feasibility of a packaging-based, behaviorally informed intervention aimed at improving antibiotic adherence among urinary tract infection patients in a low-resource setting.

Data on patient flow at each stage were collected to estimate recruitment, retention, and compliance rates.

The intervention was designed to minimize cognitive load, enabling patients with varying literacy levels to participate without additional counseling.

As a pilot study, the sample size was not powered to test clinical effectiveness, limiting conclusions to feasibility outcomes.

Conducted across six tertiary care hospitals, enhancing generalizability within the province, although results may not extend to rural or private-sector settings.

**Supplementary Information:**

The online version contains supplementary material available at 10.1186/s12879-025-12246-x.

## Introduction

Antibiotic resistance (ABR) is a mounting global health emergency, with projections indicating that antibiotic–resistant infection could cause 10 million annual deaths by 2050 [1]. The misuse of antibiotics contributes significantly to the development of this resistance [2–4]. Non-adherence, in particular, results in sub-therapeutic dosing, enabling resistant pathogens to survive and multiply, thereby undermining both individual treatment outcomes and global containment efforts [5, 6]. Thus, it represents one of the most detrimental yet modifiable drivers of ABR [7, 8]. Addressing non-adherence is, therefore, a critical step toward mitigating the ABR crisis.

Recognizing this urgency, the World Health Organization has prioritized cost-effective, context-adapted behavioral interventions as essential strategies to improve antibiotic use and curb ABR [9]. However, in low- and middle-income countries, most intervention studies conducted so far heavily rely on digital platforms such as Short Message Service reminders or mobile applications [10–12]. While these can be effective, they remain inaccessible to large segments of the population living in low-resource settings. For example, mobile phone penetration in Pakistan is still below 50% [13]. This digital divide underscores a pressing need for scalable, non-digital, and low-cost interventions that can be seamlessly integrated into routine care.

Beyond digital exclusion, structural constraints within healthcare delivery also hinder effective adherence counseling. In many developing countries, average consultation times are less than five minutes, and below three minutes in Pakistan [14], leaving minimal opportunity for the physician to educate the patient regarding the significance of completing antibiotic courses. The issue is further compounded by community pharmacies that often operate without pharmacists or have pharmacists whose role often tends to be merely dispensing medications without counseling [15–18]. These challenges highlight the need for interventions that do not rely on extended consultation time or depend on professional oversight and place negligible cognitive burden on patients.

Nudge theory, proposed by Thaler and Sunstein (2008), stated that subtle modifications in the way choices are presented can influence people’s decisions and behaviors without restricting their freedom of choice or significantly changing economic incentives [19]. Extending this concept, embedding visual storytelling into medication packaging provides a highly scalable, low-cost and non-digital solution to antibiotic non-adherence. By integrating simple pictorial cues into routine medication taking, such packaging transforms an ordinary pill container into a continuous behavioral cue that minimizes cognitive effort, accommodates individuals with varying literacy levels, and facilitates patient understanding even when consultation time is brief or counseling is absent. This approach would be feasible in settings with limited smartphone access, requires no additional infrastructure, and integrates seamlessly into existing pharmacy and clinical workflows, directly aligning with the World Health Organization’s priority for developing context-sensitive, cost-effective behavioral interventions to combat ABR [9].

Uncomplicated urinary tract infections (UTIs) could serve as an appropriate clinical model for evaluating behavioral interventions for rational antibiotic use in low-resource settings, as they are highly prevalent in lower–middle–income countries and contribute to considerable morbidity and healthcare costs [9]. They are usually treated with short, standardized antibiotic regimens that provide a well-defined period for adherence to antibiotics [20]. However, in Pakistan, more than half of patients prematurely discontinue therapy, often when symptoms improve or due to socioeconomic constraints, undermining treatment success and accelerating resistance [6, 21]. This is especially concerning as *Escherichia coli*, the predominant uropathogen, has shown resistance rates exceeding 50% to first-line agents such as ciprofloxacin and ceftriaxone [21, 22].

In this context, we designed a pilot randomized controlled trial (RCT) to assess the feasibility of implementing a culturally adapted, nudge-based visual storytelling intervention to support antibiotic adherence among UTI patients. The study evaluated feasibility at both the trial level (screening, eligibility, recruitment, prescription filling, and retention rates) and intervention level (fidelity of sticker delivery). In addition, antibiotic adherence was measured as an exploratory outcome to generate preliminary effect size estimates and inform the design and sample size calculation for a future definitive trial.

## Method

This pilot RCT was designed and reported following the CONSORT 2010 extension for randomized pilot and feasibility trials [23].

### Trial design and setting

**Fig. 1:**
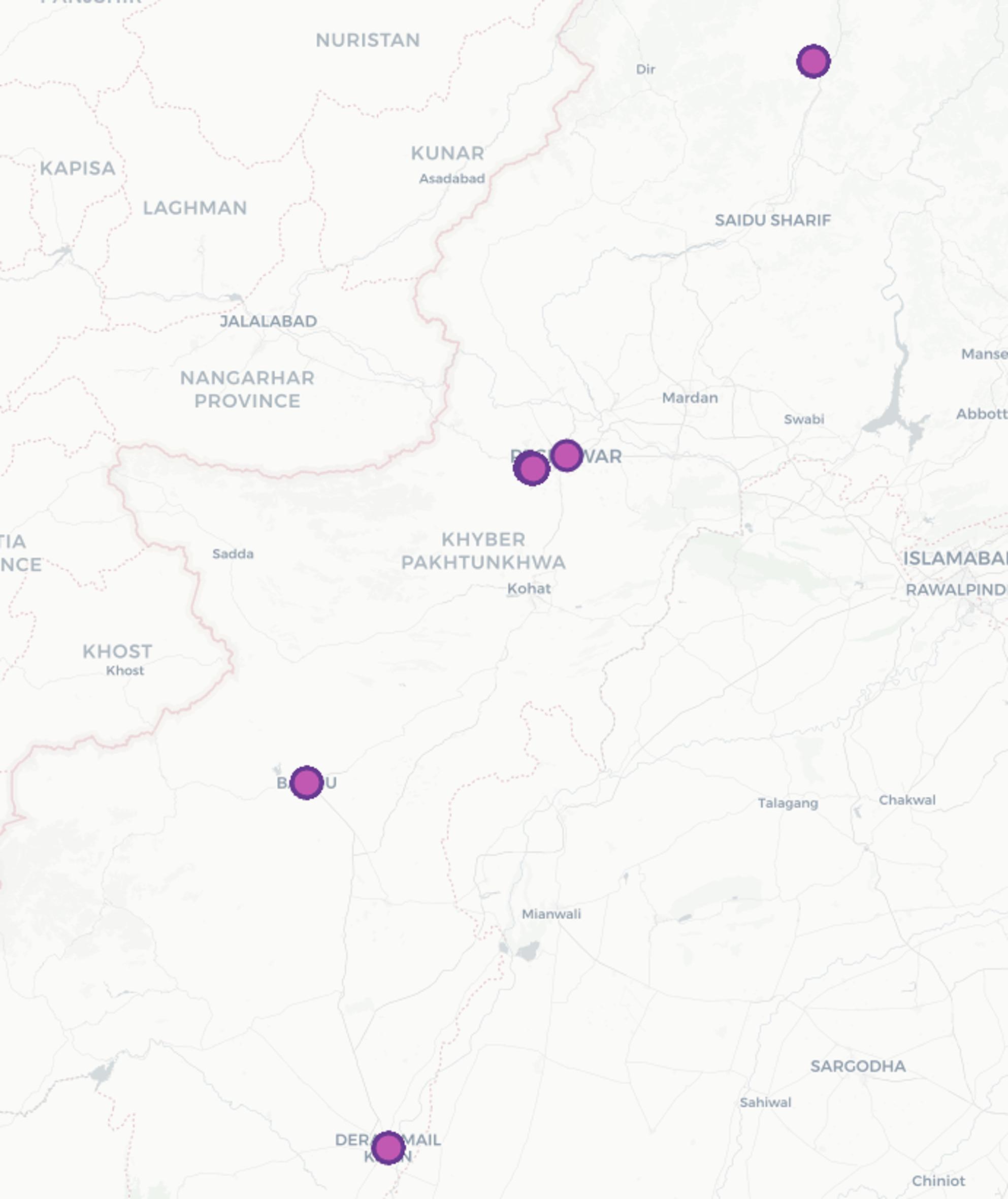
Selected study areas. Map source: Epicollect5 [28]

This pilot RCT employed a parallel two-arm design. Participants were randomly allocated to either the intervention group (visual storytelling sticker) or the control group (standard care) in a 1:1 ratio. The trial was conducted in outpatient urology/nephrology departments of six tertiary care hospitals in Khyber Pakhtunkhwa, Pakistan (Peshawar, *n* = 3, Swat, *n* = 1, Bannu, *n* = 1, Dera Ismail Khan, *n* = 1). The selected study areas are given in Fig. 1. The study was conducted between May 2025 to July 2025. 

### Participants

The participants in the current study were physician-confirmed patients with uncomplicated UTIs. The inclusion criteria for the patients were: (1) physician-confirmed uncomplicated UTI diagnosis, (2) prescribed oral antibiotic therapy (tablets or capsules), (3) obtained their prescribed medication, and (4) were willing to participate. Exclusion criteria encompassed patients with polypharmacy (concurrently using ≥ 5 medications), complicated UTIs (e.g., pyelonephritis, sepsis, or structural abnormalities), multiple comorbidities, cognitive impairment, or pregnancy/lactation status. These exclusions minimized confounding factors related to complex treatment regimens, clinical severity, and physiological variables that could influence adherence behavior. Participants were screened during outpatient visits using the SEAR (Screened, Eligible, Approached, Randomized) framework to ensure protocol compliance [24].

### Sample size

As this study was a pilot trial, we did not perform a formal sample size calculation aimed at hypothesis testing. Instead, our sample size was determined with the primary purpose of estimating feasibility outcomes with adequate precision and to generate preliminary effect size estimates for planning a future definitive RCT. We planned for recruiting 30 participants for each arm (total *n* = 60), consistent with recommendations from methodological recommendations for pilot trials [25–27], which suggests sample sizes of approximately 12 to 35 participants per arm for pilot trials are typically sufficient to provide stable estimates of key feasibility parameters and to inform sample size calculation for the definitive trial, while maintaining an appropriate balance between methodological rigor and resource efficiency.

### Intervention

**Fig. 2:**
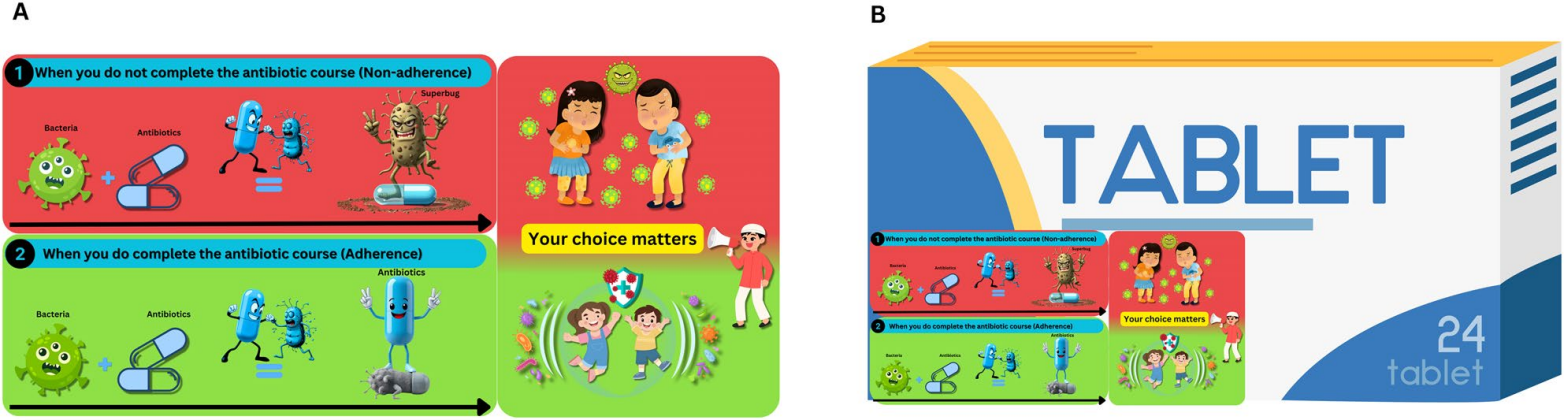
Intervention sticker depicting a visual storytelling approach to antibiotic adherence (**A**) and Application of the intervention sticker integrated onto the antibiotic box (**B**)

The intervention group received a culturally adapted visual storytelling sticker affixed to their antibiotic packaging, as shown in Fig. 2. This sticker was developed through a theory-driven, multi-step process: First, it was grounded in Nudge theory and structured using the Taxonomy of Choice Architecture and MINDSPACE (Messenger, Incentives, Norms, Defaults, Salience, Priming, Affect, Commitments, Ego) framework. The sticker featured two contrasting panels: (1) A red panel depicting non-adherence consequences, showing bacteria evolving into “superbugs” when antibiotics are stopped prematurely. (2) A green panel illustrating adherence benefits, where complete antibiotic courses eradicate bacteria.

The intervention was developed and validated through an expert consensus (40 experts; 3 rounds; ≥70% agreement) and refined via a patient understanding study (*n* = 30), which confirmed 93.3% comprehension of the core message. The sticker (2.5 × 6 cm) was applied during medication dispensing at hospital/pharmacy counters, ensuring visibility without obscuring critical drug information.

The control group received standard care, which consisted of verbal or written instructions about antibiotic use from healthcare providers, consistent with routine clinical practice in the study setting.

### Randomization

Randomization was conducted separately for each participating hospital using block randomization with a fixed block size of six to maintain balanced allocation between groups. A unique randomization list for each hospital was generated in R using the “blockrand” package by an independent researcher (I.H) who was not involved in participant recruitment or intervention delivery. To ensure allocation concealment, the randomization sequence was utilized using sequentially numbered, opaque, sealed envelopes (SNOSE), which were opened only after a participant was deemed eligible and consented. Recruitment and allocation were monitored weekly to ensure group balance.

### Implementation

The intervention was delivered by affixing the visual storytelling stickers to antibiotic packs at the time of dispensing, either at hospital or pharmacy counters. Data collectors underwent standardized training prior to initiation of the study, which covered correct sticker application, accurate pill-count procedures, and ethical engagement with participants to ensure informed and respectful interactions. To maintain fidelity to the intervention protocol, weekly audits were conducted throughout the study period to verify consistent and correct application of the stickers and adherence to data collection procedures.

### Blinding

Due to the nature of the behavioral intervention, blinding of participants and site staff delivering the intervention was not feasible. However, the physician who diagnosed the patients and the data analyst were blinded. The data analysis was blinded by neutrally labeling the groups as “A” and “B”.

### Outcome

The primary outcome was feasibility, assessed at both trial and intervention levels. The trial–level feasibility metric includes: (1) screening rate, (2) eligibility rate, (3) recruitment rate, (4) prescription filling rate, and (5) retention rate, whereas the intervention–level metric includes intervention delivery fidelity (Number pack with correct sticker application/total audited). Antibiotic adherence was measured as an exploratory outcome to inform the design of future trials, specifically sample size calculations. It was assessed at the end of the prescribed course using pill counts and calculated as the proportion of pills taken relative to the total prescribed. This measure was not used to evaluate the effectiveness of the intervention.

### Data collection

Data were collected using EpiCollect5, a secure, cloud-based platform for mobile data collection that facilitates real-time monitoring and centralized management [28]. Initially, the screening and enrollment data were collected from the patients through trained data collectors. Subsequently, baseline patient demographics, multidimensional poverty, and prescription data were collected from the included patients. At the initial follow-up, the patient’s adherence data were collected during a hospital revisit or a household visit.

### Statistical analysis

Feasibility metrics were reported as proportions with 95% confidence intervals computed using Wilson’s method. Baseline characteristics were summarized as means with standard deviations for continuous variables and as frequencies with percentages for categorical variables. Groups were compared using independent samples t-tests for continuous variables and Chi-square or Fisher’s Exact tests (*n* < 5 expected cell counts) for categorical variables. Antibiotic adherence (an exploratory outcome) was analyzed solely to estimate effect size variability for sample size calculation. Adherence proportions were calculated per group, and the absolute difference, along with 95% confidence intervals, was reported. A complete-case analysis was used for all outcomes (no imputation), as missing data directly inform the feasibility assessment for the definitive trial. All analyses were conducted using the R statistical software version 4.5.1.

### Study ethics

The Ethics Committee of the Department of Pharmacy Practice, Bahauddin Zakariya University, Multan (Approval No. BZU FOPDPP 2466) and Saidu Group of Teaching Hospital, Swat (Approval No. 1401/03) reviewed and approved the study. This pilot trial was not prospectively registered, as it primarily aimed to assess feasibility and generate preliminary estimates for a definitive trial. The definitive trial has been prospectively registered at ClinicalTrials.gov (Identifier: NCT06885658), and this pilot study represents an ethically approved preparatory step toward that registered trial.

All participants provided written informed consent in the local language. For participants who were unable to read, consent was obtained after the study information was read aloud, and then they signed the consent form. Participants were informed that their participation was voluntary and that they could withdraw at any time. Participant confidentiality was maintained throughout the study. The participants were treated in accordance with the Declaration of Helsinki.

## Results

### Participants’ flow and feasibility metrics

**Fig. 3:**
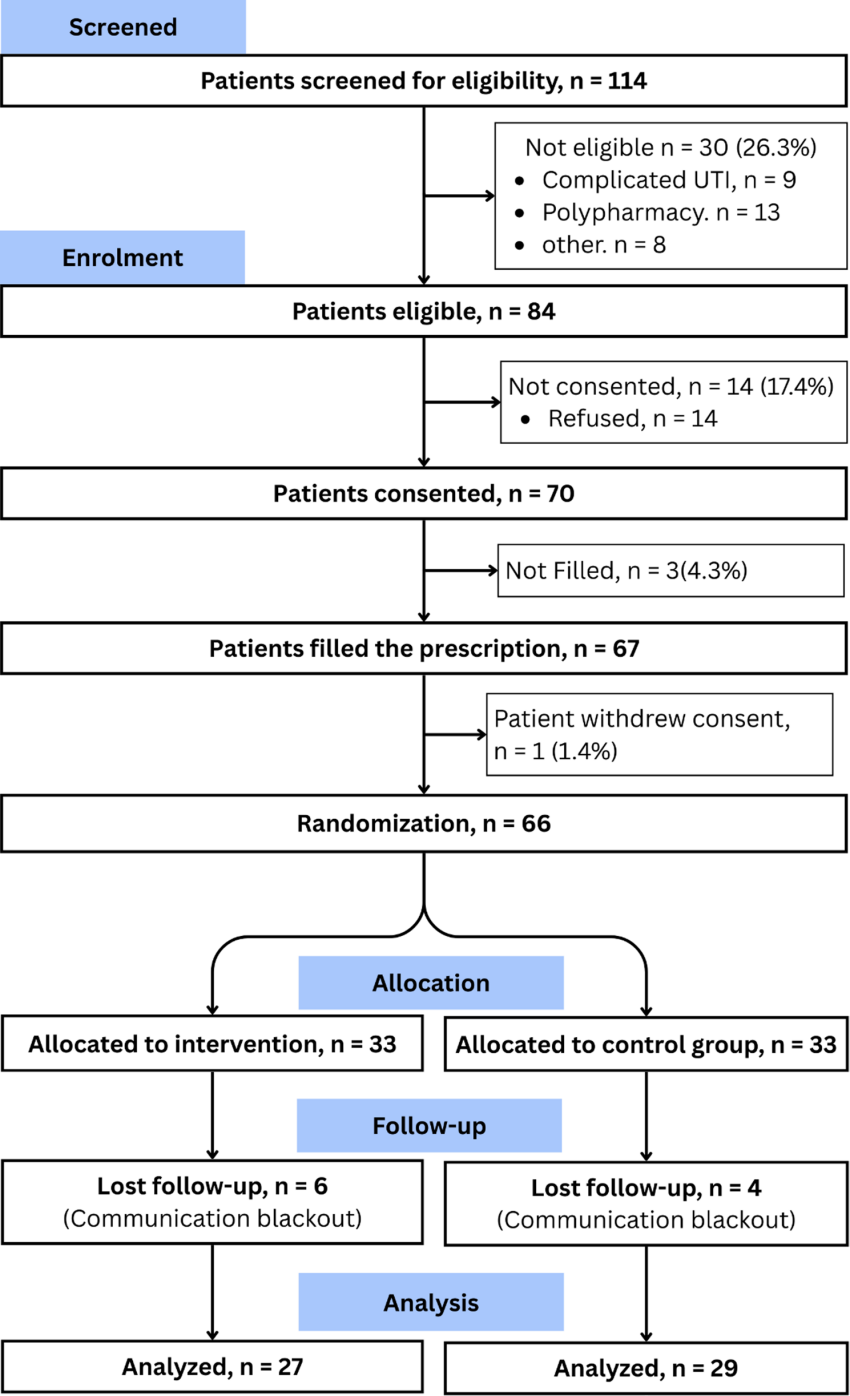
CONSORT flow diagram for the pilot RCT

A total of 120 patients were approached for participation, and 114 patients were screened. Of these, 30 patients (26.3%) did not meet the inclusion criteria, primarily due to polypharmacy (*n* = 13) and complicated UTI (*n* = 9). Eighty-four patients were eligible, of whom 70 provided written informed consent, yielding a recruitment rate of 61.4% (95% CI: 52.2–69.8). Following consent, three patients (4.3%) did not fill their prescriptions, and one patient (1.4%) withdrew consent prior to randomization. Sixty-six patients were randomized in equal allocation to the intervention (*n* = 33) and control groups (*n* = 33). During follow-up, six patients (18.2%) from the intervention group and four participants (12.1%) from the control group were lost to follow-up, mainly due to communication blackouts in the two conflict zones (Dera Ismail Khan and Bannu). Overall, 56 participants completed the study and were included in the final analysis, corresponding to a retention rate of 84.8% (95% CI: 74.3–91.6). Intervention delivery fidelity was assessed in a subsample of 16 patients in the intervention arm and confirmed correct delivery in 14 patients, resulting in a fidelity rate of 87.5% (95% CI: 64.0–96.5). The details are presented in Fig. 3; Table 1.

**Table 1:** Assessment of the feasibility metrics

Trial–level feasibility	Point estimate (%)	95%CI
Screening rate (114/120)	95.0	89.5–97.7
Eligibility rate (84/114)	73.7	64.9–80.9
Recruitment rate (70/114)	61.4	52.2–69.8
Prescription filling rate among consented (60/70)	95.0	92.0–99.7
Retention rate (56/66)	84.8	74.3–91.6
Per–arm retention		
Intervention (27/33)	81.8	65.6–91.4
Control (29/33)	87.9	72.7–95.2
**Intervention level feasibility**		
Intervention delivery fidelity (14/16)	87.5	64.0–96.5

### Baseline data

Baseline characteristics of the 56 analyzed participants are presented in Table 2. The intervention and control groups were well-balanced in terms of age (*p* = 0.42), gender (*p* = 0.59), education (*p* = 0.48), employment status (*p* = 0.41), residential status (*p* = 0.75), and poverty level (*p* = 0.71). A notable imbalance was observed in marital status (*p* = 0.001), with the control group containing substantially more divorced/widowed patients compared to the control group (62.1% vs. 14.8%).

**Table 2:** Baseline characteristics of the patients (*n* = 56)

	Intervention *n* = 27	Control *n* = 29	*P*-value
	*n* (%)	*n* (%)	
**Age** (mean ± SD)	45.7 ± 16.35	49.59 ± 19.37	0.42
18–30	7 (25.9)	7 (24.1)	
31–40	3 (11.1)	2 (6.9)	
41–50	7(25.9)	5 (17.2)	
51–60	4 (14.8)	5 (17.2)	
> 60	6 (22.2)	10 (34.5)	
**Gender**			0.59
Male	13 (48.1)	16 (55.2)	
Female	14 (51.1)	13 (44.8)	
**Education**			0.48
No formal education	6 (22.2)	4 (13.8)	
Primary education	7 (25.9)	13 (44.8)	
Secondary education	10 (37)	7 (24.1)	
>secondary education	4 (14.8)	5 (17.2)	
**Employment**			0.41
Unemployed	16 (59.3)	14 (48.3)	
Employed	11 (40.7)	15 (51.7)	
**Marital status**			0.001
Unmarried	9 (33.3)	2 (6.9)	
Married	14 (51.9)	9 (31.0)	
Other	4 (14.8)	18 (62.1)	
**Residential status**			
Rural	11 (40.7)	13 (44.8)	0.75
Urban	16 (59.3)	16 (55.2)	
**Poverty**			0.71
Not deprived	5 (18.5)	8 (27.6)	
Vulnerable	5 (18.5)	3 (10.3)	
Deprived	6 (22.2)	5 (17.2)	
Severely deprived	11 (40.7)	13 (44.8)	

SD- standard deviation, Other – divorced, widowed

### Preliminary adherence outcome

**Fig. 4:**
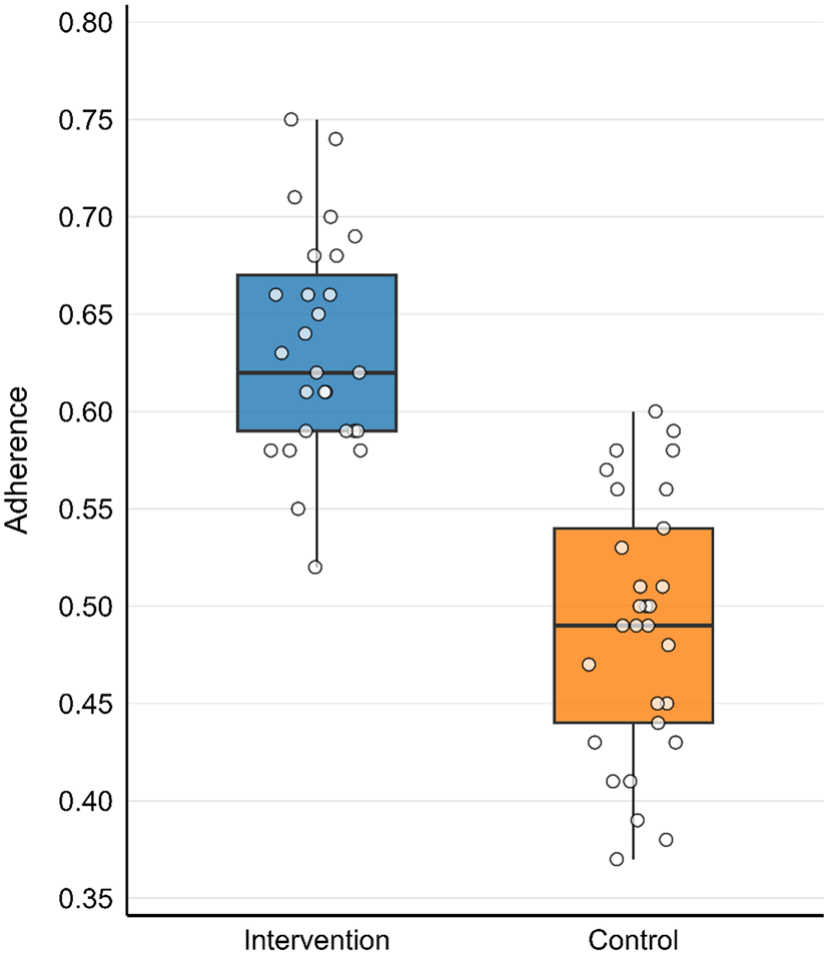
Boxplot comparing adherence between control and intervention groups

The preliminary adherence outcome showed a 14.3% (95%CI 11.0% to 17.6%) improvement in the intervention group compared to the control group, as shown in Fig. 4. This analysis indicated a potential difference between groups; however, given the small sample and pilot design, these findings are not definitive and should be interpreted only as preliminary estimates to inform sample size calculations for a larger trial.

## Discussion

This pilot RCT aimed to examine the feasibility of implementing a nudge-based visual storytelling sticker to improve antibiotic adherence among patients with uncomplicated UTI. In keeping with the CONSORT extension for pilot and feasibility studies, our focus was on feasibility rather than on establishing efficacy [29, 30]. The results are encouraging, particularly given the sociocultural and health system challenges in this region. Recruitment of 61.4% of eligible patients demonstrates that participation in a behavioral trial is feasible, even in busy tertiary hospitals where outpatient clinics are often overcrowded and have short consultation times. Retention of 84.8% exceeded the 80% benchmark typically used to judge feasibility [26], despite difficulties such as conflict-related communication blackouts in districts like Dera Ismail Khan and Bannu. The intervention delivery fidelity of 87.5% further demonstrates that the intervention sticker is easy and feasible to integrate into dispensing practice with minimal disruption, a finding that aligns with fidelity thresholds recommended for behavioral interventions [31]. Taken together, these findings provide strong evidence that both the trial methods and the intervention were feasible in the context of a resource-limited healthcare system, supporting progression to a larger definitive trial.

An imbalance in marital status was observed, with a higher proportion of divorced or widowed participants in the control group. This pattern most likely reflects the effects of chance variation in a small pilot trial, where even a few participants can disproportionately affect group distributions [26]. The use of block randomization without stratification further increases the likelihood of such imbalances, as allocation balances group size but not participant characteristics. Moreover, because analyses were restricted to participants who completed follow-up, post-randomization attrition may have accentuated the apparent imbalance. While marital status is not a pre-specified determinant of adherence, it may influence treatment behavior indirectly through differences in social support or socioeconomic vulnerability, particularly for widowed or divorced women in the cultural context of the region. To minimize this risk in the definitive trial, we will adopt stratified randomization to ensure balanced allocation. These measures will reduce the potential for confounding and improve the internal validity of the trial.

Although this study was not powered for hypothesis testing, we observed a 14% absolute difference in adherence favoring the intervention group. This exploratory finding should be interpreted cautiously, but its magnitude is consistent with previous RCTs and systematic reviews reporting improvements of approximately 10–15% when visual or pictorial cues were incorporated into medication use [32–34]. Evidence also suggests that pictorial communication can help overcome barriers linked to low health literacy [35], which is highly relevant in Pakistan’s context. Unlike these prior studies, however, our trial was explicitly designed as a pilot to establish feasibility of the intervention, where no similar feasibility work has been conducted. These findings therefore provide preliminary effect size estimates to guide sample size calculations and inform the design of a future definitive RCT.

Several important lessons were learned for the design of a future large-scale trial. First, while recruitment and retention were satisfactory, improvements can be made by incorporating multiple contact strategies to further minimize attrition, especially in remote areas. Second, although intervention fidelity was high, occasional lapses underscored the need for ongoing training of intervention delivery staff and regular fidelity checks. Third, an imbalance in marital status between trial arms illustrates the vulnerability of small pilot trials to random variation; in the definitive trial, stratified randomization strategies will be applied to ensure balanced allocation. Fourth, adherence was measured using pill counts, which was a pragmatic choice in this setting but is known to have limitations, as patients may discard tablets rather than consume them. More sophisticated strategies, such as electronic monitoring or pharmacy refill tracking, are currently unavailable or not feasible in Pakistan, particularly in public-sector hospitals. As such, pill count remains the most practical and widely used approach in this context.

This study has several limitations. The sample size was small and not intended to provide definitive evidence of effectiveness; observed outcome differences may have been due to chance. Neither participants nor intervention delivery staff were blinded to allocation, raising the possibility of performance bias. However, to minimize this, an independent researcher, who had no role in the intervention delivery and data collection, created the randomization list and shared it with field staff. The pill-count adherence measure, while objective, may overestimate adherence in some cases. A structural limitation of this trial is the inability to obtain the full denominator of all UTI consultations during the study period. The participating tertiary-care hospitals use paper-based outpatient records with no electronic diagnostic registry, and the very high patient volume made it operationally infeasible to record every UTI diagnosis across parallel clinics. Consequently, the screening log reflects only those patients directly assessed by the research team, and some selection bias cannot be excluded. Despite these limitations, the pilot achieved its primary aim of establishing feasibility, while also highlighting key methodological refinements required for a larger trial.

## Conclusion

This pilot randomized trial showed that a packaging-based visual storytelling sticker can be feasibly incorporated into antibiotic dispensing in tertiary hospitals in Pakistan. The study achieved satisfactory recruitment, retention, and intervention fidelity thresholds. Although not powered to determine effectiveness, exploratory findings indicated a possible improvement in adherence, offering preliminary estimates to aid sample size calculations. Overall, these findings support moving forward to a definitive multicenter randomized trial to thoroughly assess the intervention’s impact on antibiotic adherence and its potential role in fighting antimicrobial resistance.

## Supplementary Information

Below is the link to the electronic supplementary material.

Supplementary Material 1

## Data Availability

All data generated or analyzed during this study are included in this published article.

## Abbreviations

ABR: Antibiotic resistance
UTI: Urinary tract infection
RCT: Randomized controlled trial
SEAR: Screened, Eligible, Approached, Randomized
MINDSPACE: Messenger, Incentives, Norms, Defaults, Salience, Priming, Affect, Commitments, Ego
